# What Can Genome-Wide Association Studies Tell Us about the Genetics of Common Disease

**DOI:** 10.1371/journal.pgen.0040033

**Published:** 2008-02-29

**Authors:** Mark M Iles

**Affiliations:** University College London, United Kingdom

## Abstract

The success of genome-wide association studies relies on much of the risk of common diseases being due to common genetic variants; but evidence for this is inconclusive. The results of published genome-wide association studies are examined to see what can be learnt about the distribution of disease-associated variants and how this might influence future study design. Although replicated disease-associated variants tend to be very common and frequency is inversely correlated with estimated effect size, our simulations suggest that such observations are the result of power. We find that for studies conducted to date, the frequency and effect size of significantly associated alleles are likely to be similar to those of the underlying disease alleles that they represent. Little of the genetic variation of disease has been explained so far, but current studies are only adequately powered to detect very common alleles unless they greatly increase disease risk. Thus, although the truth of the common disease / common variant hypothesis remains undecided, recent successes suggest that there are many more common genetic disease-associated variants, requiring larger studies to be identified.

## Introduction

In the last year there has been a dramatic increase in the publication of the results of genome-wide association (GWA) studies. The timing reflects recent technological improvements in genotyping technology, but the impetus behind these studies can be traced back to two key papers from 1996 [1,2]. These two papers argued that common variants may underlie many common diseases, that these would be more easily found using population-based association studies rather than family-based linkage analysis even if this required testing every gene in the genome [1], and that all common variants in human genes should be identified [2]. These proposals gained credence and led to the International HapMap Project [3], with the aim of cataloguing common human genetic variants. Combined with the latest SNP chip genotyping technologies allowing the simultaneous genotyping of hundreds of thousands of markers, HapMap has enabled GWA studies to be conducted, leading to the recent discovery of common genetic variants associated with diseases such as coronary heart disease [4–8], breast cancer [9–11] and type II diabetes [12–18].

GWA studies require the collection of large numbers of cases with a particular disease and controls, genotyped at many markers across the genome. As a result of the association (linkage disequilibrium, or LD) between alleles at nearby loci, not all loci in a region need be typed for the majority of common variation to be captured. Marker (usually single nucleotide polymorphism or SNP) spacing should be dense enough to capture the variation at those loci that have not been genotyped. SNPs may be chosen randomly across the genome or may be chosen specifically for their coverage (using a pilot sample or existing data such as HapMap) in which case they are known as tagging SNPs [19]. Studies should be designed in terms of both sample size and marker coverage to have sufficient power to detect common disease susceptibility alleles of modest effect. Genotype data may be analysed in various ways, but the simplest is a comparison of frequencies between cases and controls, often using the Cochrane-Armitage trend test, which assumes a multiplicative risk model. Power issues will be discussed in more depth later.

GWA represents a method for capturing a new class of disease-associated genetic variants. Pedigree-based association studies utilise families in which disease clusters, and so are powered to find rare variants of large effect. GWA meanwhile relies on population-based samples, and so requires common variants (as rarer variants will be unobserved) of more modest effect, which could not be found using traditional linkage-based approaches.

### 

#### Common disease/common variant hypothesis.

The common disease / common variant (CDCV) hypothesis assumes that much of the genetic variation of a common complex disease is due to relatively few common variants. If multiple rare genetic variants were the primary cause of common complex disease, association studies would have little power to detect them; particularly if allelic heterogeneity existed. Ironically, given the recent huge financial and scientific investment in GWA, there is not a great deal of evidence in support of the CDCV hypothesis and much of it is equivocal. The first evidence was that common genetic variants had been found to increase the risk of some common diseases, such as APOE which increases risk for Alzheimer's and heart disease [20], and that many had been convincingly replicated [21]. But there also exist examples of rare variants influencing common disease [22,23], telling us that both rare and common variants may influence common diseases, although we do not know which is more important. The second source of evidence came several years later from theoretical population genetic models. Unfortunately, any conclusions depend greatly on the model used, for which many of the variables are unknown or at least difficult to estimate [24–27].

Despite such limited and sometimes contradictory evidence supporting the CDCV hypothesis, GWA studies have proved very popular. Their success at uncovering many common alleles associated with common disease suggests that the hypothesis is true to an extent, at least for some diseases studied, but it is useful to look at the results in more depth. GWA studies are said to represent an “agnostic” [28] approach to identifying the genetic variants that influence common human diseases, being “unbiased by prior assumptions about the DNA alterations responsible” [29]. Thus, the results of published GWA studies may include valuable information about the genetic basis of common diseases, especially the CDCV hypothesis, as in [21]. The more that is known about the underlying genetic basis of human disease the better studies can be designed to identify those genetic variants that influence human diseases.

The simplest approach is to examine the distribution of the frequency of those disease-associated alleles found by GWA studies and subsequently confirmed. But this does not account for rarer variants being harder to detect or disease-associated alleles having different frequencies from the causative alleles they tag. For this reason, we simulated data to see what underlying distributions could give rise to the observed frequency distribution of significantly associated alleles. These simulations were used to estimate correlations between factors such as marker frequency and effect size, and the frequencies of the most significant marker allele and the disease allele.

### Proportion of genetic risk

While disease-susceptibility variants found using pedigree-based linkage analysis tend to have large relative risks, they have little effect on disease risk at a population level, due to their rarity. More common genetic variants, despite having only moderate disease risk, may be far more important in terms of public health simply because they are more common. Many GWA reports have included estimates of the influence of the genetic variants found on population-level disease risk, using various methods. We discuss how the methods vary and how these estimates may be interpreted, as the proportion of risk explained may influence future study design.

#### Findings from genome-wide analyses.

The results of 54 studies across 22 different diseases (Table S1) were examined. Most were GWA analyses, while some followed up the results of GWA analyses. Only those SNPs found initially in a GWA study (excluding the few SNPs that were already known, such as those in the Major Histocompatibility Complex) and that reached nominal significance in at least one other study were included. This gave 45 disease-associated SNPs. Almost all had reached genome-wide significance (*p* < 5 × 10^−7^ as in [6]) and been replicated in at least one independent population. Two SNPs did not reach this level of significance in a single study but had a *p*-value of at least 10^−5^ in two independent studies. The estimated allele frequency and odds ratio (OR) (preferably from follow-ups to reduce bias) were recorded for each confirmed disease-associated SNP. In summarizing the data, SNPs associated with age-related macular degeneration [30] and Exfoliation Glaucoma [31] were ignored, as their estimated odds ratios are very high and they were detected with small sample sizes, making them both outliers that tend to skew the results from the remaining 43 SNPs.

**Figure 1:**
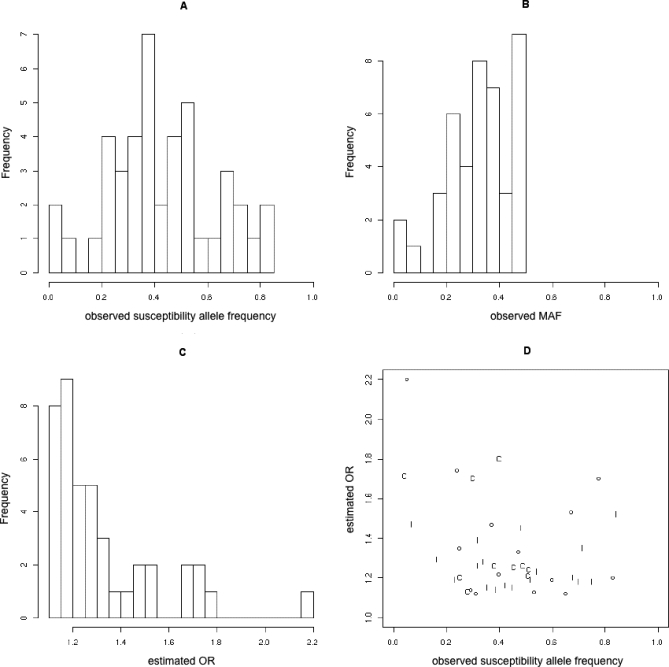
Allele Frequencies and ORs Observed in GWA Studies (A and B) Histograms of susceptibility allele frequency and MAF, respectively, at confirmed susceptibility loci. (C) Histogram of estimated ORs at confirmed susceptibility loci. (D) Plot of estimated OR against susceptibility allele frequency at confirmed susceptibility loci. “I”s represent SNPs associated with autoimmune disease, “C”s represent SNPs associated with cancer, and small circles represent SNPs associated with other diseases.

The distribution of disease-associated allele frequencies (Figure 1A) looks reasonably Normal, despite the small number of observations, with a median frequency of 0.40 (95% CI: 0.37, 0.48) and a mean of 0.43 (95% CI: 0.39, 0.49). Only three of the 43 SNPs have minor allele frequency (MAF) <0.1 (Figure 1B). This suggests that most of the alleles associated with common diseases are common. The distribution of estimated ORs (Figure 1C) is skewed with a median of 1.25 (95% CI: 1.2, 1.29). Only eight of the 43 SNPs have an OR >1.5 and only one of these an OR >2. Superficially, these results suggest that most disease-associated alleles are fairly common, but this does not account for power. Susceptibility alleles where the MAF is high are far more easily detected.

Pearson's correlation between susceptibility allele frequency and OR was −0.28 (95% CI: −0.53,0.07, *p* = 0.07) (Figure 1D). A negative correlation between effect size and frequency may be expected due to selection pressures [27]. However, the stronger negative correlation when MAF is studied instead of susceptibility allele frequency (−0.48: 95% CI: −0.66, −0.19, *p* = 0.001) suggests that some of this correlation is due to power. As allele frequency tends towards the extremes (0 and 1), power will decrease so only large effects will be found. There is no need to invoke selection to explain this observation.

Thus, apparent patterns in the findings may be explained by power considerations. We investigated these potential problems by simulation.

#### Simulation of genome-wide analyses.

Realistic case/control data were simulated utilising the ENCODE data (http://www.hapmap.org) assuming a single ungenotyped disease susceptibility locus, based on realistic frequency distributions [26,32] (Figure S1) with mutation rates chosen to give disease alleles that were either almost exclusively low frequency (*β_S_* = 0.1), mostly low frequency but some more frequent (*β_S_* = 1), or mainly higher frequency (*β_S_* = 3) (Figure S2). *n* = 1,000 or *n* = 3,000 cases and controls were produced with genotype relative risks (GRRs) of 1.2, 1.5, and 2. Genotyped SNPs were selected to mimic those on a SNP chip. The Cochrane-Armitage trend test was applied to all “genotyped” SNPs and the *p*-value for genome-wide significance set at *α* = 5 × 10^−7^ [6]. See Text S1 for more details of simulations. Ideally, we would hope that the distributions of significant marker locus frequencies are distinguishable at different mutation rates.

**Figure 2:**
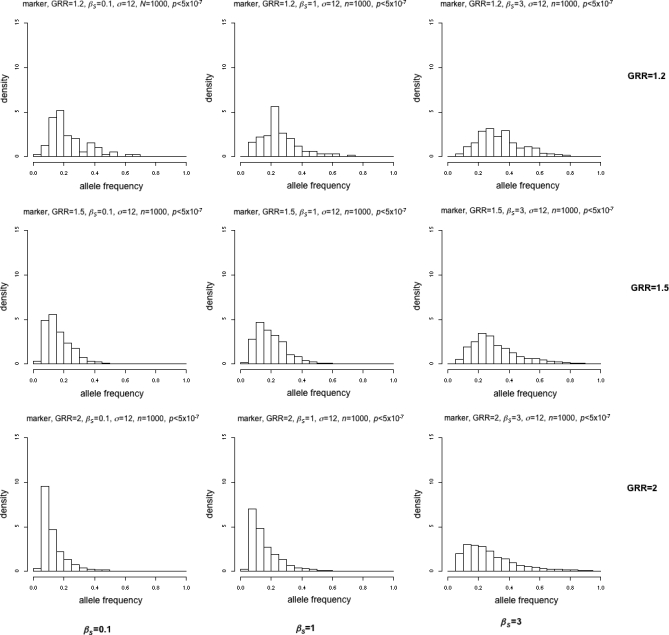
Simulations Showing Frequencies of Disease-Susceptibility–Related Loci Found with *p* < 5 × 10^−7^ and a Sample Size of 1,000 Rows are (from top to bottom) GRR = 1.2, 1.5, 2; columns are (from left to right) *β_S_* = 0.1, 1, 3.

**Figure 3:**
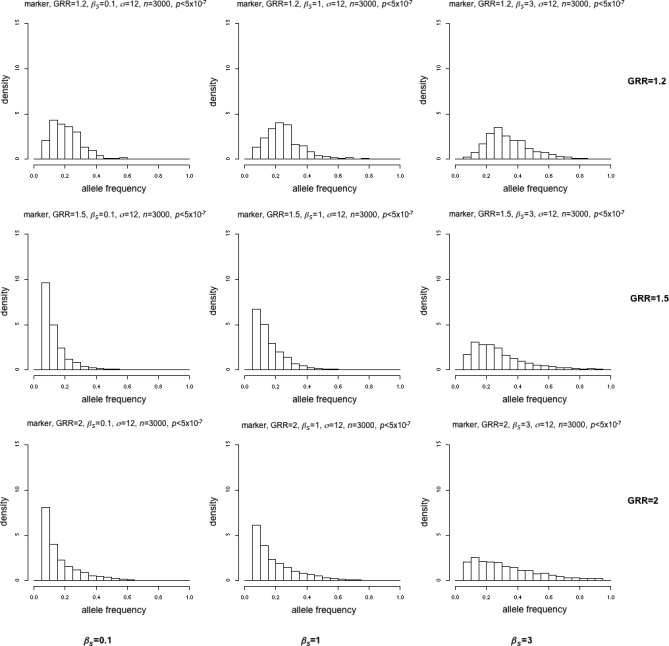
Simulations Showing Frequencies of Disease-Susceptibility–Related Loci Found with *p* < 5 × 10^−7^ and a Sample Size of 3,000 Rows are (from top to bottom) GRR = 1.2, 1.5, 2; columns are (from left to right) *β_S_* = 0.1, 1, 3.

The different distributions are easily discernable when GRR = 2 (Figures 2 and 3), even for *n* = 1,000 (Figure 2). However, when the GRR = 1.5, the distributions are quite similar for *n* = 1,000 (Figure 2). Only when *n* = 3,000 is there a clear increase in rare variants for *β_S_* = 0.1 or 1 (Figure 3). When GRR = 1.2 (close to the median of the observed GRRs in GWA studies), the distributions for *β_S_* = 0.1, 1, and 3 are extremely similar for both *n* = 1,000 and 3,000.

Thus, whatever the underlying distribution of disease variant frequencies, the results suggest that unless the effect size or sample size is large (GRR > 1.5 or *n* = 3,000), simulations with mostly rare (*β_S_* = 0.1) or common (*β_S_* = 3) susceptibility alleles produce similar distributions of disease-associated allele frequencies that look Normal and not too skewed, with a median of 0.2–0.4. These results seem to hold for modes of inheritance other than multiplicative (Text S2, Figures S3–S7).

The correlation between GRR and MAF ranged from −0.33 to −0.51—a negative correlation between GRR and allele frequency of similar magnitude to that of the real data, though none was simulated.

For *n* = 1,000 and GRR = 1.2, 1.5, and 2, correlations between marker and susceptibility allele frequency were 0.63, 0.92, and 0.83, respectively. For *n* = 3,000, correlations for GRR = 1.2, 1.5, and 2, were 0.91, 0.85, and 0.62, respectively. Thus, unless both sample size and effect size are large, the correlation is strong. The frequency of the disease-associated allele at the marker locus is thus a good indicator of the frequency of the genuine disease allele at the susceptibility locus under the model used here.

The absolute difference between estimated GRR and the true, simulated GRR was examined. The average difference for GRR = 1.2, 1.5, 2 was 0.31, 0.14, 0.19 for *n* = 1,000 and 0.08, 0.08, 0.32 for *n* = 3,000. Thus, GRR estimates are likely to be fairly reliable. It is interesting that estimates of both disease allele frequency and GRR are generally less reliable as power increases (either through greater sample size or GRR). This is likely to be because when power is high, markers that are in weaker LD with the causative locus may reach significance and estimates will then be less reliable. This is reflected in the fact that GRR estimates tend to overestimate for *n* = 1,000 but less so for *n* = 3,000. When *n* is larger, power is greater and SNPs in weaker LD reach significance, but their weak LD will result in lower GRR estimates. Smaller sample sizes exhibit the so-called “winner's curse”, consistently overestimating effect sizes [33,34].

We also found that even when the true disease model was dominant/recessive, the best-fitting model at the marker locus was biased slightly but consistently away from dominant/recessive towards a multiplicative risk model (Text S2). This suggests that even when the true mode of inheritance is strongly dominant or recessive, the apparent mode of inheritance at the most significant marker locus is biased towards multiplicative. These results also provide further support for using a test, such as the Cochrane-Armitage trend test, that assumes a multiplicative risk model.

Our models assume a single disease variant in each region. If there are multiple disease variants, the results may be somewhat different. Nor have we considered very rare causative alleles. It would be hoped that such effects would not greatly affect the conclusions.

#### Power considerations.

These results suggest that the distribution of frequencies for confirmed disease-associated alleles is far more reflective of power than of the underlying distribution of disease alleles. Given that the case/control samples for GWA usually number in the thousands and are gradually increasing, it might be expected that such studies are well-powered. However, several papers have shown that, given the strict genome-wide significance criteria that studies must fulfill, power is much less than might be imagined [35,36].

**Figure 4:**
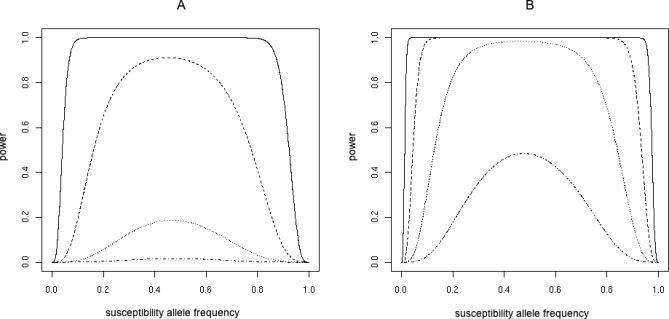
Power Calculated Using Quanto for 1,000 Cases and Controls (A) and 3,000 Cases and Controls (B) Lines are for GRR = 2 (solid), 1.5 (dashed), 1.3 (dotted), 1.2 (dashed and dotted). A multiplicative mode of inheritance is assumed.

Power estimates produced in Quanto [37,38], assuming all variants have been typed with a multiplicative mode of inheritance, show that there is good power to detect a variant with a GRR of 2 even at low frequency (Figure 4). A variant with a GRR of 1.5 is detectable down to a MAF of 0.05 for *n* = 3,000, but only has decent power for MAF > 0.2 for *n* = 1,000. For GRR = 1.3, power is good at high frequency (MAF > 0.2) for *n* = 3,000, but generally poor (power < 0.2) whatever MAF for *n* = 1,000. For GRR = 1.2, power is poor even for *n* = 3,000. These power calculations may seem dispiriting, given that the median observed GRR is about 1.2. In fact, these results are optimistic. The calculations assume that the disease locus itself has been genotyped, when in fact it is more likely to be a nearby SNP in incomplete LD. Given problems of overfitting, incomplete marker ascertainment [39], population differences, SNP failure (6.2% in [6]), and uneven spacing, there are many sources causing overestimation of coverage. If there are multiple susceptibility variants interacting epistatically (so that their marginal effects are weak) power will be further reduced.

The effect of coverage (measured by r^2^) on power is best understood by knowing that to detect an ungenotyped variant using a genotyped SNP, the sample size must be increased by a factor of 1/r^2^ compared to the sample size required when testing the variant itself [40]. A disease locus whose effect is detectable when genotyped with a sample of *n* = 1,000 will require *n* = 1,250 if a nearby SNP is instead genotyped with an r^2^ = 0.8 between the two. Power estimates from our simulations bear these results out and show the effect of using tagging SNPs (Figure S2).

Reports of coverage are often reported as the proportion of known SNPs captured by typed markers with r^2^ > 0.8. While a useful shorthand for comparison, it is a gross simplification—disease SNPs captured with r^2^ < 0.8 may still be captured, but with less power. It should be remembered that choosing 0.8 as the cutoff for coverage is quite arbitrary, as is using *p* = 0.05 as a cutoff for significance in hypothesis testing.

Despite low power, disease-associated SNPs have been found. The power distribution also suggests that those variants that have been found are the most common and so the easiest to detect. Few associated variants have a frequency below 0.2, but the limited power at these frequencies for GRRs < 1.5 suggests that they may represent only a fraction of the existing disease variants. Estimating how much of the overall risk known variants explain may be useful.

#### Estimating the risk explained.

Another way of looking at risk is to estimate how much of the (genetic) risk is explained by known genetic variants. Some studies claim their findings explain much of the variation in disease risk, but the methods used differ and the findings are variable. Population attributable risk (PAR) estimates the effect of a factor on incidence: if that factor were removed from the population, by how much would incidence fall? Other measures estimate the proportion of genetic variance or excess familial risk explained by a variant, a more direct measure of the known proportion of overall genetic risk. If a susceptibility allele is very common in the population, say with a frequency close to 1, it is likely to have an important effect on disease risk and will have a high PAR, even if its effect on risk is small, because in its absence general disease risk will fall. However, it will make very little contribution to variation in disease risk whatever its effect size because it is so common.

Reported PARs tend to be high, as the variants are common: 0.54 for Restless Legs Syndrome [41], 0.38 for Coronary Artery Disease [7], and 0.13 for Prostate Cancer [42]; while measures of the proportion of the genetic risk are lower: excess familial risk of 0.036 for Breast Cancer [11] and 0.002 of the variance in risk for Multiple Sclerosis [43]. Tellingly, estimates of PAR for the replicated SNP found for colorectal cancer vary between 0.11 and 0.42 (because of differences in frequency between populations), while explaining only 0.009–0.018 of the increased risk to siblings of cases [44]. It is also well known that initial estimates are likely to be greatly overestimated [34,45]. For several diseases, such as Parkinsons disease [46], bipolar disorder [6,47], and hypertension [6], no new replicable variant has yet been found using GWA.

Misunderstanding PAR may give the impression that for several reported diseases, most of the underlying genetic cause has been identified. In fact, the variants found to date are likely to represent only a small proportion of the overall variation in disease risk [29]. It is likely that there are other common variants to be found (if the CDCV hypothesis is true), and that many rare variants also have an effect [48] but will be far more difficult to detect.

## Discussion

It may be convenient to assume that the genetic variants underlying common diseases are themselves common, simply because that is what has been observed to date. However, our results show that such an assumption would be naïve and potentially misleading.

Through simulation of common disease, we have shown that for the size of studies carried out so far and the effect size of the variants found, we would expect any significantly associated alleles to be common even when the causative genetic variants are mostly rare. For *n* = 1,000, this result changes only if the effect size is large (GRR = 2) (Figure 2), and even for *n* = 3,000 the frequency distributions of significant alleles are very similar for rare and common causative genetic variants unless the effect size is quite large (GRR = 1.5) (Figure 3). At the smallest effect size (GRR = 1.2, the closest to the median observed in reality), there is little to distinguish distributions even at sample sizes of 3,000 cases and controls (Figure 3).

Simulations show a strong correlation between the frequency of the disease-associated allele and the causative allele. Thus, common marker variants associated with disease represent similarly common variants directly causing disease, demonstrating that common variants certainly exist.

Estimates of the genetic variation explained suggest that even for those diseases where common genetic variants have been found, most genetic variation is still to be uncovered. This does not imply that the CDCV hypothesis is necessarily false, rather that power is low for current study size unless MAF is high or effect size is large. Thus, while many very frequent disease variants have been found for the diseases studied so far by GWA, there may be many more variants that are of moderate frequency but that current studies are not large enough to find. We cannot yet rule out the possibility that much genetic variation is due to rare variants.

And what of the future? Sample sizes will increase, leading to greater power to find rare variants. But when samples are larger, increased power may mean that markers in weaker LD with the disease locus reach significance, if the same (or less stringent, if Bayesian) significance levels are used. Thus, as sample sizes increase, rare variants are more easily detected, but the most significantly associated individual markers may not be rare themselves. Sequencing is the only way to completely avoid this latter problem, although it only slightly improves power (Figure S2) and will not on its own remove the bias towards finding more common variants. There is likely to be a limit to how large population-based studies can get, and so there may be a further class of variants that are too rare to be captured by GWA but are not sufficiently high risk to be captured by population-based linkage (for examples see [49]). New approaches will be needed to find these, perhaps utilising bioinformatics-based methods to identify candidate genes and variants.

Many of the findings from GWA studies that have not quite reached genome-wide significance may be genuine and could be uncovered by combining the results from several studies, perhaps by meta-analysis or marker imputation if SNP panels vary [50].

For now, it is unlikely that much can be inferred about the CDCV hypothesis from the results of GWA studies. The successes in finding common variants associated with common diseases are encouraging, but, as our findings show, we cannot yet be sure whether the common disease-associated variants found so far represent the tip of the iceberg or the bottom of the barrel.

## Supporting Information

Figure S1 Distribution Function of Frequencies According to Wright's Formula When *σ* = 12, *β_N_* = 0.01 and *β_S_* = 0.1, 1, or 3 (Notation from Pritchard, 2001) Used for simulated disease allele frequency distribution, assuming that all disease alleles are at a single locus.
(439 KB TIF)

Figure S2 Estimated Power Power estimated by simulation to reach significance level of *p* = 5 × 10^−7^, grouped by frequency 0.05–0.1, 0.1–0.15, ..., 0.45–0.5 for sample sizes *n* = 1,000 and 3,000 (first and second row, respectively) and GRR = 1.2, 1.5, 2 (first, second, and third column, respectively). Circles are for Affymetrix-like coverage, crosses are for sequencing all variants with MAF > 0.05.
(2.2 MB TIF)

Figure S3 Simulations Showing Frequencies of Disease-Susceptibility-Related Loci Found with *p* < 5 × 10^−7^ and a Sample Size of 1,000 Under a Recessive Disease Model GRRs are chosen such that the population prevalence is equivalent to GRRs under a multiplicative model of GRR = 1.2, 1.5, 2. Rows are (from top to bottom), GRR = 1.2, 1.5, 2; columns are (from left to right) *β_S_* =0.1, 1, 3.
(1.8 MB TIF)

Figure S4 Simulations Showing Frequencies of Disease-Susceptibility–Related Loci Found with *p* < 5 × 10^−7^ and a Sample Size of 3,000 under a Recessive Disease Model GRRs are chosen such that the population prevalence is equivalent to GRRs under a multiplicative model of GRR = 1.2, 1.5, 2. Rows are (from top to bottom) GRR = 1.2, 1.5, 2; columns are (from left to right) *β_S_* = 0.1, 1, 3.
(1.8 MB TIF)

Figure S5 Simulations Showing Frequencies of Disease-Susceptibility–Related Loci Found with p < 5 × 10^−7^ and a Sample Size of 1,000 under a Dominant Disease Model GRRs are chosen such that the population prevalence is equivalent to GRRs under a multiplicative model of GRR = 1.2, 1.5, 2. Rows are (from top to bottom) GRR = 1.2, 1.5, 2; columns are (from left to right) *β_S_* = 0.1, 1, 3.
(2.7 MB TIF)

Figure S6 Simulations Showing Frequencies of Disease-Susceptibility–Related Loci Found with *p* < 5 × 10^−7^ and a Sample Size of 3,000 under a Dominant Disease Model GRRs are chosen such that the population prevalence is equivalent to GRRs under a multiplicative model of GRR = 1.2, 1.5, 2. Rows are (from top to bottom) GRR = 1.2, 1.5, 2; columns are (from left to right) *β_S_* = 0.1, 1, 3.
(2.7 MB TIF)

Figure S7 Estimated Power for Non-Multiplicative Modes of Inheritance Power estimated by simulation to reach significance level of *p* = 5 × 10^−7^, grouped by frequency 0.05–0.1, 0.1–0.15, ..., 0.45–0.5 for sample sizes *n* = 1,000 and 3,000 (first and second row, respectively) and GRRs chosen to give same disease incidence as a multiplicative model with GRR = 1.2, 1.5, 2 (first second and third column, respectively). Circles are for a recessive model, crosses are for dominant, both with Affymetrix-like coverage.
(2.2 MB TIF)

Table S1 Genome-Wide Association Analyses and Follow-Up Studies Used Here (47 KB DOC)

Text S1 Supplementary Text on Simulation of GWA Data (23 KB DOC)

Text S2 Looking at Non-Multipicative Modes of Inheritance (22 KB DOC)
