# Supplementary material for: What Can Genome-Wide Association Studies Tell Us about the Genetics of Common Disease
Source: PLoS Genet. 2008 Feb 29;4(2):e33. doi: 10.1371/journal.pgen.0040033 (PMC2323402; doi:10.1371/journal.pgen.0040033)
Supplement: Supplementary file 1 [file pgen.0040033.sd001.doc]

**Text S1**

**Supplementary Text on simulation of GWA data**

Data were simulated by randomly selecting one of the 10 phased ENCODE regions (http://www.hapmap.org) and choosing SNPs at random, weighting by MAF, such that their density and frequency distribution (mean density 1 SNP per 5.8kb, median distance 3.5kb, mean MAF 0.27, 96% of genome within 10kb of a SNP) were close to that used in the Affymetrix 500k array set (mean density 1 SNP per 5.8kb, median distance 2.5kb, mean MAF 0.22, 85% of genome within 10kb of a SNP) although slightly more evenly spaced across the regions. These represented the ‘genotyped’ SNPs. All SNPs with minor allele frequency less than 0.05 were then excluded from both the genotyped and ungenotyped sets as GWA studies have very little power to detect variants with low MAF.

A non-genotyped SNP was selected at random from the data to be the disease-susceptibility locus. Selection of the disease susceptibility locus was weighted by frequency to follow Wright’s formula [Wright, 1949[31]], as used in [Pritchard, 2001[26]] (Figure S1), $f\left(p\right)=kp^{\beta_{S}-1}\left(1-p\right)^{\beta_{N}-1}e^{\sigma\left(1-p\right)}$ where *p* is allele frequency, *σ* represents selection and *β_S_* and *β_N_* mutation rates to the disease and non-disease states respectively. *k* is a normalization constant. Here *σ*=12, *β_N_*=0.01, *β_S_*=0.1, 1 or 3. These values give realistic distributions with disease alleles either almost exclusively low frequency (*β_S_*=0.1), mostly low frequency but some more frequent (*β_S_*=1) or mainly higher frequency (*β_S_*=3) (Figure S2).

Case-control samples were generated by sampling with replacement entire haplotypes at random, based on the allele at the disease locus. While this could inflate LD between SNPs the ENCODE samples are large and the analyses dependent only on pairwise LD between common markers, so that little such inflation is likely. Case-control data were simulated with GRRs of 1.2, 1.5 and 2, a multiplicative mode of inheritance, a population prevalence of disease of 5% and equal numbers of cases and controls (*n*=1000 or 3000). The Cochrane-Armitage trend test was applied to all ‘genotyped’ SNPs. The observed allele frequencies and the estimated GRRs (assuming a multiplicative mode of inheritance) were recorded for the disease SNP and the most strongly associated ‘genotyped’ SNP. This procedure was repeated 10,000 times (50,000 times for GRR=1.2, *n*=1000) and the frequency and effect size distributions were examined. We restricted the results to either those that reached a significance of 5x10^-7^ as in the Wellcome Trust study [WTCCC, 2007[6]].

Correlations were calculated using only those simulations that had reached genome-wide significance (p<5x10^-7^). The correlation between marker allele frequency and estimated GRR was calculated, but as samples were simulated with fixed GRRs rather than a range of GRRs, the data were combined across GRRs to give one dataset for each sample size and each *β_S_*. This is equivalent to assuming that GRR is independent of the population model and that GRRs of 1.2, 1.5 and 2 are equally likely to occur

The correlation between the frequency of the most significant marker and the susceptibility allele was calculated and averaged over the three *β_S_* models.

Rather than calculate correlations between the estimated GRR and the true, simulated GRR we looked at the absolute difference between the two in each simulation and averaged over these. Again this is because samples were simulated with fixed GRRs. Only simulations that had reached p<5x10^-7^ were included.

The effect of sequencing rather than genotyping was investigated under the same models by assuming that every marker with MAF>0.05 had been genotyped, including the susceptibility locus. Thus we could see whether sequencing improved the discrimination of marker frequency distributions modelled with different values of *β_S_*. Power was estimated using the simulations for both genotyped and sequenced data.
