# Supplementary material for: What Can Genome-Wide Association Studies Tell Us about the Genetics of Common Disease
Source: PLoS Genet. 2008 Feb 29;4(2):e33. doi: 10.1371/journal.pgen.0040033 (PMC2323402; doi:10.1371/journal.pgen.0040033)
Supplement: Supplementary file 2 [file pgen.0040033.sd002.doc]

**Text S2**

**Looking at non-multipicative modes of inheritance**

We investigated the effect of modes of inheritance other than multiplicative. Dominant and recessive modes of inheritance were simulated with GRRs chosen such that the disease incidence would be the same (0.05) as a disease susceptibility allele with the same frequency under a multiplicative mode of inheritance. For a recessive mode of inheritance and GRR=2 this was not possible for p<0.11. This adjustment also means that power for the recessive mode of inheritance is high and for dominant low, compared to a multiplicative mode of inheritance (Figure S7). Observed frequency distributions of significantly associated alleles for different underlying disease susceptibility allele frequency distributions are similar to those for a multiplicative mode of inheritance. For a recessive model the underlying frequency distributions are slightly more distinguishable.

We also investigated how easily a non-multiplicative risk model could be detected at the most significant marker locus, given the breakdown of non-multiplicative models that is likely to occur as a result of incomplete LD. We simulated multiplicative and dominant risk models (as above) and fitted multiplicative and dominant models to each simulated susceptibility locus and most significant marker locus. For each simulation at both the susceptibility and marker locus we recorded whether the best-fitting model was multiplicative or dominant if either reached significance of p<5x10^-7^ at the marker locus. We then compared how often the best model was multiplicative at the susceptibility locus (of those that reached significance) with how often the best model was multiplicative at the marker locus (of those that reached significance). Those models with less than 1% power were excluded.

Unsurprisingly, there were fewer significant simulations at the marker locus than at the susceptibility locus, and the proportion that were best fitted by a multiplicative model, when this was the model simulated, was virtually the same at both the disease and marker locus (median of 0.99% fewer at the marker locus). When the model simulated was dominant, those simulations that reached significance were more likely to be multiplicative at the marker locus than the disease locus (this was true for all dominant models, with a median increase of 6.8%)

This suggests that a model of risk based on the most significant marker is slightly biased towards a multiplicative risk model, even when the underlying risk model at the susceptibility locus is strongly dominant. This may also indicate that statistical tests of markers may be more powerful when assuming a multiplicative risk model (as in the Cochrane-Armitage trend test) than a more general model.
