# Supplementary material for: What Can Genome-Wide Association Studies Tell Us about the Genetics of Common Disease
Source: PLoS Genet. 2008 Feb 29;4(2):e33. doi: 10.1371/journal.pgen.0040033 (PMC2323402; doi:10.1371/journal.pgen.0040033)
Supplement: Supplementary file 3 [file pgen.0040033.st001.doc]

**Table S1.** Genome-wide association analyses and follow-up studies used here.

| Disease | Studies |
| --- | --- |
| Age-related macular degeneration | Klein et al. (2005) Science 308: 385-389.  Zareparsi et al. (2005) Am. J. Hum. Genet. 77: 149-153. |
| Parkinson's | Maraganore et al. (2005) Am. J. Hum. Genet. 77: 685-693.  Clarimon et al. (2006) Am. J. Hum. Genet. 78: 1082-1084.  Farrer et al. (2006) Am. J. Hum. Genet. 78: 1084-1088.  Goris et al. (2006) Am. J. Hum. Genet. 78: 1088-1090.  Li et al. (2006) Am. J. Hum. Genet. 78: 1090-1092. |
| Adult and childhood obesity | Herbert et al. (2006) Science 312: 279-283. |
| Wet Age-related macular degeneration | DeWan et al. Science 314: 989-992.  Yang et al. Science 314: 992-993. |
| Crohn's disease | Duerr et al. (2006) Science 314: 1461-1463.  Hampe et al. (2007) Nat. Genet. 39: 207-211.  WTCCC (2007) Nature 447: 661-678.  Parkes et al. (2007) Nat. Genet. 39: 830-832.  Libioulle et al. (2007) PLoS Genet. 3: e58.  Rioux et al. (2007) Nat. Genet. 39: 596-604.  Raelson et al. (2007) Proc Natl Acad Sci USA 104:14747-14752 |
| Type II diabetes | Sladek et al. (2007) Nature 445: 881-885.  Saxena, R. et al. (2007) Science 316: 1331-1336.  Zeggini et al. (2007) Science 316: 1336-1341.  Scott et al. (2007) Science 316: 1341-1345.  WTCCC (2007) Nature 447: 661-678.  Frayling et al. (2007) Science 316: 889-894.  Steinthorsdottir et al. (2007) Nat. Genet. 39: 770-775.  Salonen et al. (2007) Am. J. Hum. Genet. 81: 338-345. |
| Type I diabetes | WTCCC (2007) Nature 447: 661-678.  Todd et al. (2007) Nat. Genet. 39: 857-863.  Hakonarson et al. (2007) Nature 448: 591-594. |
| Bipolar Disorder | WTCCC (2007) Nature 447: 661-678.  Baum et al. Mol. Psych. published online 8 May 2007 (doi:10.1038/sj.mp.4002012). |
| Rheumatoid Arthritis | WTCCC (2007) Nature 447: 661-678. |
| CHD/Stroke | McPherson et al. (2007) Science 316: 1488-1491.  Helgadottir et al. (2007) Science 316: 1491-1493.  WTCCC (2007) Nature 447: 661-678.  Samani et al. (2007) N. Engl. J. Med. 357: 443-453.  Matarin et al. (2007) Lancet Neurol. 6: 414-420. |
| Hypertension | WTCCC (2007) Nature 447: 661-678. |
| Prostate Cancer | Gudmundsson et al. (2007a) Nat. Genet. 39: 631-637.  Yeager et al. (2007)Nat. Genet. 39: 645-649.  Haiman et al. (2007a) Nat. Genet. 39: 638-644.  Gudmundsson et al. (2007b) Nat. Genet. 39: 977-983. |
| Colorectal Cancer | Tomlinson et al. (2007) Nat. Genet. 39: 984-988.  Zanke et al. (2007) Nat. Genet. 39: 989-994.  Haiman et al. (2007b) Nat. Genet. 39: 954-956. |
| Coeliac Disease | van Heel et al. (2007) Nat. Genet. 39: 827-829. |
| Breast Cancer | Hunter et al. (2007) Nat. Genet. 39: 870-874.  Stacey et al. (2007) Nat. Genet. 39: 865-869.  Easton et al. (2007) Nature 447: 1087-1095. |
| Human Gallstone Disease | Buch et al. (2007) Nat. Genet. 39: 995-999. |
| Late-onset Alzheimers | Grupe et al. (2007) Hum. Mol. Genet. 16: 865-873. |
| Restless Legs | Stefansson et al. (2007) N Engl J Med 357(7): 639-647.  Winkelmann et al. (2007) Nat. Genet. 39:1000-1006. |
| Childhood Asthma | Moffatt et al. (2007) Nature 448: 470-473. |
| Multiple Sclerosis | International Multiple Sclerosis Genetics Consortium (2007) N. Engl. J. Med. 357(9): 851-862  Gregory et al. (2007) Nat Genet 39(9): 1083-1091  Lundmark et al. (2007) Nat Genet 39(9): 1108-1113 |
| ALS | Schymick et al. (2007) Lancet Neurol 6:322-328  Dunckley et al. (2007) N Engl J Med 357(8):775-788  van Es et al. (2007) Lancet Neurol 6(10):869-877 |
| Glaucoma | Thorleifsson et al. (2007) Science 317:1397-1400 |
